# Genetically Boosting Electron Transfer in Electroactive Biofilms for Improved Sensitivity of Microbial Fuel Cell‐Based Biosensing

**DOI:** 10.1111/1751-7915.70356

**Published:** 2026-04-21

**Authors:** Yutong Zhang, Xi Han, Yongguang Jiang, Yiran Dong, Liang Shi, Hongqing Yin, Yidan Hu

**Affiliations:** ^1^ Department of Biological Sciences and Technology School of Environmental Studies, China University of Geosciences Wuhan China; ^2^ The Key Laboratory of Water and Sediment Sciences College of Environmental Sciences and Engineering, Peking University Beijing China; ^3^ State Key Laboratory of Biogeology and Environmental Geology China University of Geosciences Wuhan China; ^4^ Enshi Tujia and Miao Autonomous Prefecture Academy of Agricultural Sciences Enshi China

**Keywords:** cyclic GMP‐AMP, electroactive biofilms, extracellular electron transfer, *Geobacter* species, microbial fuel cell‐based biosensor

## Abstract

Electroactive biofilms (EABs) hold significant potential for applications in bioenergy, biosensing and wastewater treatment; however, their performance is frequently hindered by inefficient extracellular electron transfer (EET). A notable example of this limitation is the significant impact of low EET efficiency on the sensitivity of EAB‐based biosensors. To address this, we engineered *Geobacter sulfurreducens* anode biofilms by enhancing intracellular levels of cyclic GMP‐AMP (cGAMP) through the overexpression of cGAMP synthase. This approach resulted in the formation of thin yet highly conductive biofilms. When utilized in a microbial fuel cell biosensor, the engineered biofilm demonstrated a 20‐fold improvement in detection limit for Cd(II), achieving 0.03 mg L^−1^ compared to 0.6 mg L^−1^ for the wild‐type strain. Furthermore, mechanistic studies that integrated RNA sequencing and quantitative analysis revealed an increased expression of *c*‐cytochromes and nanowires while simultaneously reducing exopolysaccharide production, thereby enhancing EET and biosensor sensitivity. Given the widespread role of cGAMP across *Geobacter* spp., dominant microorganisms in natural EABs, this study presents a broadly applicable strategy for optimizing EABs to advance technologies based on these systems.

## Graphical abstract

Engineering *Geobacter* biofilms through the manipulation of the cyclic GMP‐AMP signalling system led to the formation of thin, highly conductive bioanodes. This boosted extracellular electron transfer, enabling a microbial fuel cell biosensor with a 20‐fold lower Cd(II) detection limit (0.03 mg L^−1^) by increasing cytochromes/nanowires and reducing exopolysaccharides.

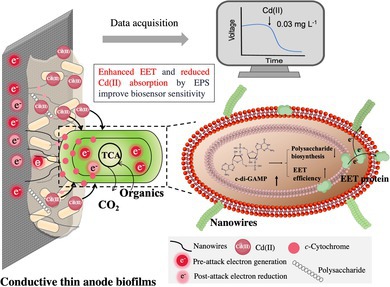

## 1 Introduction

Electroactive biofilms (EABs) are microbial communities composed of electroactive bacteria that can directly exchange electrons with electrodes (ter Heijne et al. 2021). This unique capability, mediated by extracellular electron transfer (EET), enables diverse applications, including bioenergy generation (e.g., microbial fuel cells and MFCs) and early warning systems for water biotoxicity, as well as environmental remediation efforts such as pollutant degradation (You et al. 2023) (He et al. 2022; Liu et al. 2025). Despite this potential, the inherently low EET efficiency of naturally formed EABs critically hinders their practical performance across these applications. A notable example is MFC‐based biosensors, where the bioanode functions as both a sensing and transducing element. In these systems, toxic compounds often inhibit microbial activity in the anode EAB, leading to a measurable decrease in electrical output (Prévoteau and Rabaey 2017; Hu et al. 2022). The limited EET efficiency of EAB often restricts the ability to generate comparable electrical signals in the presence of analytes, thereby affecting the sensitivity of MFC‐based biosensors.

Current strategies to enhance EET include genetically engineering model electroactive bacteria, particularly high‐current‐producing *Geobacter* species. An established strategy is to overexpress genes responsible for flagellar assembly or exopolysaccharide production in *Geobacter sulfurreducens* , which enhances biofilm formation and subsequently improves overall EET efficiency (Liu et al. 2019; Zhuang et al. 2020). Given that EET across *Geobacter* biofilms fundamentally depends on *c*‐cytochromes (*c*‐Cyts) and nanowires for short‐ and long‐distance electron transfer to electrodes (Reguera et al. 2005; Shi et al. 2016), a more direct approach is to engineer these components to enhance EET within individual cells as well as throughout the biofilm matrix. This is exemplified by several successful interventions: overexpressing nanowire‐associated genes (*pilA*, *omcZ*, *omcS* and *omcT*) increases current generation (Wang et al. 2023); enhancing the expression of the extracellular cytochrome OmcS elevates EET efficiency (Malvankar et al. 2012); and in situ engineering of histidine‐tagged pili leads to the formation of conductive histidine: nickel biohybrid structures, thereby directly reducing electron transfer resistance in *G. sulfurreducens* biofilms (Wang et al. 2024). While effective, these interventions remain limited by their focus on single‐gene or pairwise protein modifications, neglecting systemic regulatory mechanisms.

A promising alternative to enhance EET involves the modulation of cyclic dinucleotide signalling, particularly through cyclic di‐GMP (c‐di‐GMP) and cyclic GMP‐AMP (cGAMP), which globally regulates the expression of EET machinery. Our recent studies have demonstrated that reduced levels of c‐di‐GMP lead to an upregulation in nanowire gene expression, thereby improving EET in both electrode‐respiring and mineral‐respiring *G. sulfurreducens* biofilms (Hu et al. 2024; Xu et al. 2025). Notably, cGAMP exerts a broader regulatory influence on exoelectrogenesis within *Geobacter* species (Nelson et al. 2015). Investigations have revealed that intracellular cGAMP impacts multiple genes whose protein products may facilitate electron transfer from the cytoplasmic membrane to the extracellular environment via the periplasmic space and outer membrane in *G. sulfurreducens* (Hallberg et al. 2016; Xu et al. 2025). Thus, compared with EET gene modifications, our approach leverages cGAMP signalling to orchestrate a broad, systems‐level upregulation of the EET machinery via a single genetic modification, offering a more holistic and efficient strategy.

The objective of this study was to enhance EET efficiency in *G. sulfurreducens* and to develop a sensitive MFC‐based biosensor. Specifically, we utilized an engineered strain of *G. sulfurreducens* with elevated levels of cGAMP in MFCs and assessed the performance of the MFC for biosensing applications using Cd(II) as a model toxic pollutant. Furthermore, we elucidated the mechanisms underlying the observed improvements in performance through RNA sequencing analysis and quantitative measurements.

## 2 Results and Discussion

### 2.1 The Engineered *G. sulfurreducens* Biofilms Exhibit High EET Efficiency

**FIGURE 1:**
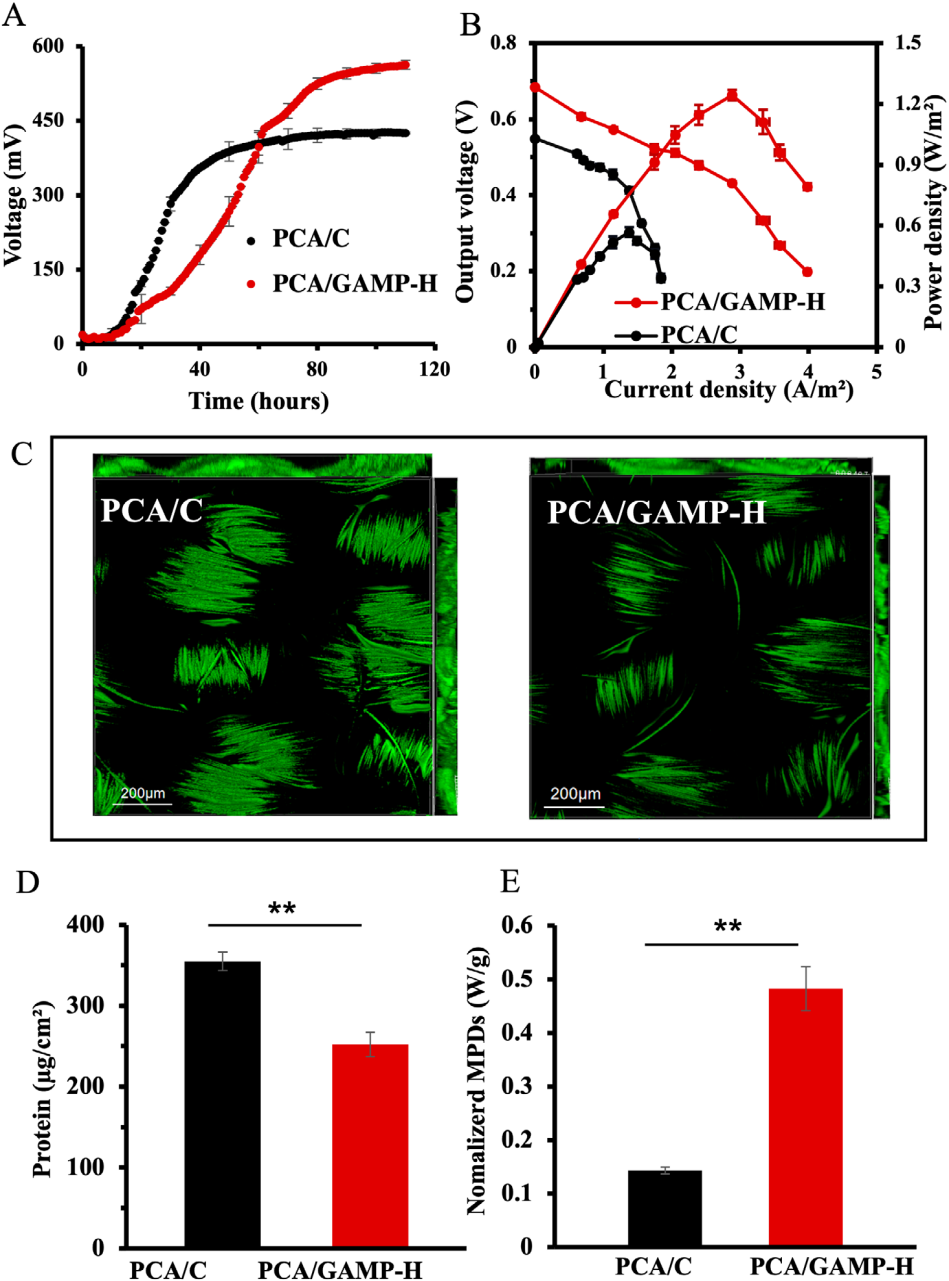
Electrochemical and biofilm analyses of *G. sulfurreducens* strains. (A) Voltage output profiles of MFCs inoculated with the cGAMP‐increased strain PCA/GAMP‐H and the control strain PCA/C with empty vector. (B) Polarization curves (left axis) and corresponding power density curves (right axis) of MFCs. (C) Representative CLSM images of anode biofilms. (D) Biomass quantified from anode‐attached biofilms. (E) Biomass‐normalized maximum power density. Data are mean ± SD (*n* = 3 independent samples). Two‐sided Student's *t*‐test was used in (D) and (E) to analyse the statistical significance (**p < 0.01).

Our previous study showed that biofilms formed by the strain PCA/GAMP‐H, which contains elevated intracellular cGAMP levels, exhibited a faster ferric mineral reduction rate compared to the control strain PCA/C that contains the empty vector (Xu et al. 2025) (Table S1). To investigate how increased cGAMP impacts EET in electrode‐respiring *G. sulfurreducens* biofilms, we compared current generation by PCA/GAMP‐H and PCA/C biofilms on MFCs anodes. The PCA/GAMP‐H strain generated a significantly higher maximum voltage output (563.4 ± 8.9 mV) than the control PCA/C strain (425.3 ± 1.8 mV; Figure 1A). Polarization and power density curves further revealed that PCA/GAMP‐H MFCs exhibited a substantially lower polarization curve slope (Figure 1B) and a higher maximum power density (1.22 ± 0.04 W m^−2^ vs. 0.51 ± 0.03 W m^−2^ for PCA/C; Figure 1B). Furthermore, electrochemical impedance spectroscopy (EIS) analysis confirmed this enhanced performance, showing PCA/GAMP‐H reduced interfacial charge transfer resistance by ~2.07‐fold compared to PCA/C (Figure S1), consistent with the polarization curve observations (Figure 1B). We also examined the redox reaction kinetics at the cell‐electrode interfaces by performing cyclic voltammetry (CV) at a low scan rate (1 mv s^−1^). The current density of PCA/GAMP‐H was significantly higher than thar of PCA/C, which demonstrated the enhanced electroactivities of PCA/GAMP‐H biofilms (Figure S2). Collectively, these results demonstrate significantly improved EET efficiency in PCA/GAMP‐H anode biofilms.

At peak voltage output, anode biofilms were stained with fluorescent viability dyes and imaged using confocal laser scanning microscopy (CLSM). The PCA/GAMP‐H strain formed a thinner biofilm (61.43 ± 2.24 μm) compared to the PCA/C control strain (82.18 ± 4.37 μm, *p* < 0.01; Figure 1C), consistent with observations in ferric mineral (Xu et al. 2025). Quantification of biofilm biomass via total protein measurement confirmed that the biomass of PCA/GAMP‐H biofilms (252.0 ± 14.9 μg cm^−2^) was approximately 1.4‐fold lower than that of PCA/C (354.9 ± 11.5 μg cm^−2^; Figure 1D). When maximum power density (MPDs) were normalized to biofilm biomass, the biomass‐normalized MPD for PCA/GAMP‐H MFCs was approximately 5‐fold higher than that for PCA/C MFCs (Figure 1E). Additionally, based on the maximum current output derived from polarization curves, the maximum electron flux through biofilms and individual cells was estimated. The maximum electron transfer rate from biofilms to the solid electrode was higher for PCA/GAMP‐H (2.48 × 10^15^ electrons s^−1^) than for PCA/C (0.99 × 10^15^ electrons s^−1^; Table S2). The single‐cell electron transfer rate increased from approximately 1.93 × 10^6^ electrons s^−1^ (PCA/C) to 6.81 × 10^6^ electrons s^−1^ (PCA/GAMP‐H). Moreover, the measured conductivity of PCA/GAMP‐H biofilms was 19.30 ± 0.56 μS/cm, which is significantly higher (*p* < 0.05) than that of the PCA/C control biofilms (15.84 ± 0.11 μS/cm). These findings consistently demonstrate significantly improved EET efficiency at both biofilm and single‐cell levels in PCA/GAMP‐H anode biofilms.

### 2.2 The Engineered *G. sulfurreducens* Biofilm Improved the Sensitivity of MFC‐Based Biosensing

To enhance the sensitivity of MFC‐based biosensors, previous studies have primarily focused on improving EET in EABs by either increasing the abundance of electroactive bacteria in microbial communities or incorporating conductive media (Qi et al. 2021). Genetically engineering EABs to facilitate electron transfer represents a promising alternative strategy, offering inherent stability and heritability. To evaluate whether the engineered thin, conductive biofilm enhances MFC‐based biosensor sensitivity, we comparatively analysed concentration‐response curves of MFCs inoculated with PCA/GAMP‐H or PCA/C following exposure to Cd(II). Cadmium is a hazardous metal that is widely distributed in wastewater, thus serving as an established model toxicant for biosensor research (Kim et al. 2007; Kumar et al. 2017; Yi et al. 2019).

**FIGURE 2:**
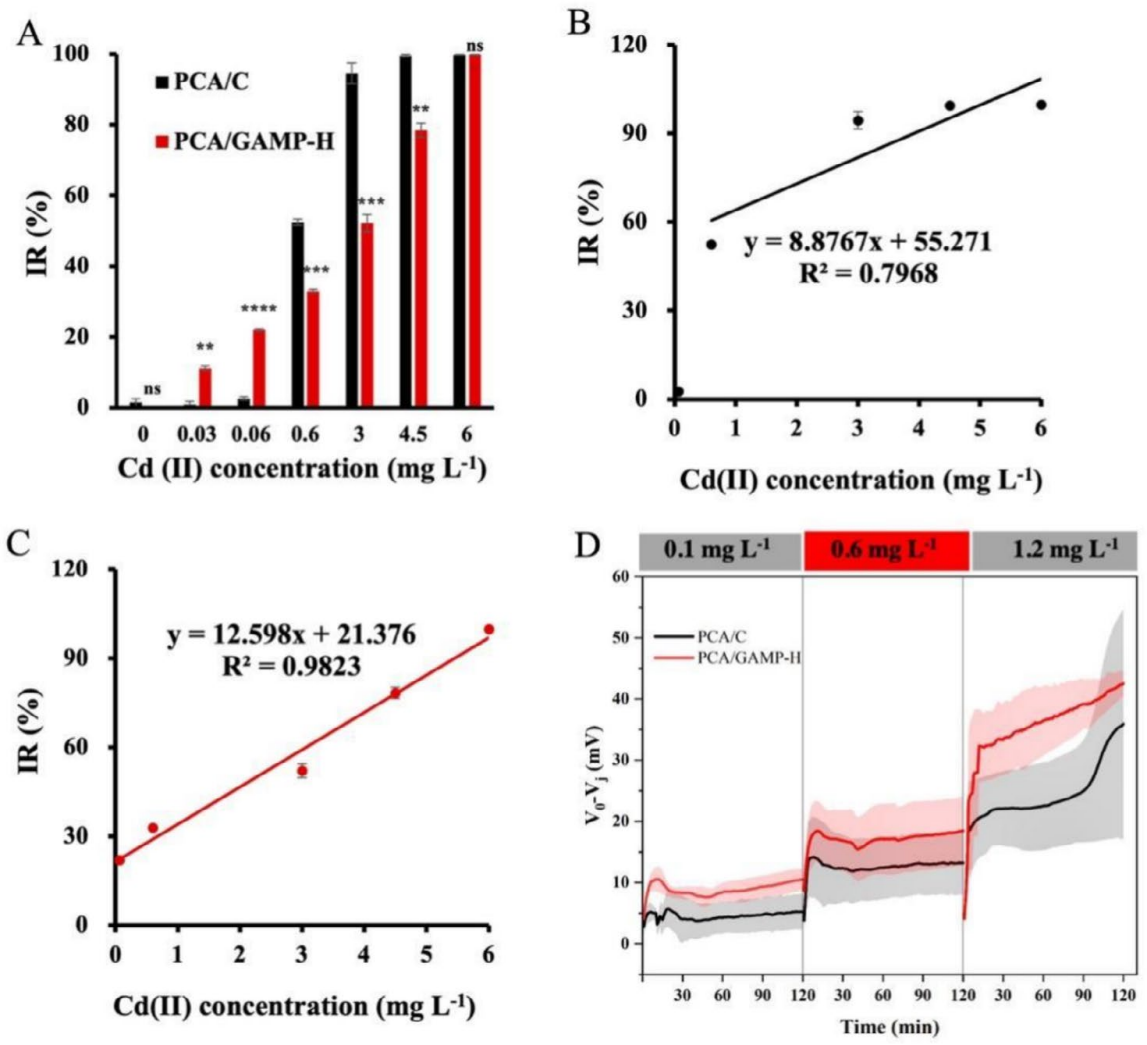
The engineered *G. sulfurreducens* biofilm as a sensing element improves the sensitivity of MFC sensors for the detection of Cd(II). (A) The IRs of the MFCs inoculated with control strain PCA/C and cGAMP‐increased strain PCA/GAMP‐H upon exposure to varying concentrations of Cd(II). (B) Fitting lines between Cd(II) concentrations and IRs for PCA/C MFCs and (C) for PCA/GAMP‐H MFCs. (D) Responses of PCA/C and PCA/GAMP‐H biosensors to Cd(II) during three consecutive sensing cycles with stepwise Cd(II) exposure at 0.1, 0.6 and 1.2 mg/L. Data are mean ± SD (*n* = 3 independent samples). Two‐sided Student's *t*‐test was used in (A) to analyse the statistical significance (**p < 0.01, ***p < 0.001; **** *p* < 0.0001; ns: no significance).

After achieving stable voltage outputs, MFCs were exposed to Cd(II) concentrations ranging from 0 to 6 mg L^−1^. Voltage outputs recorded post‐Cd(II) addition are shown in Figure S3. Cd(II) concentrations exceeding 0.6 mg L^−1^ caused a marked voltage decrease in both PCA/C and PCA/GAMP‐H MFCs within 15 min. Notably, only PCA/GAMP‐H MFCs exhibited significant voltage declines at the lower concentrations of 0.03 and 0.06 mg L^−1^. Inhibition rates (IRs) were determined 60 min post‐exposure. At low Cd(II) concentrations (0.03 and 0.06 mg L^−1^), PCA/GAMP‐H biofilm‐based sensors exhibited IRs of 11.12% ± 0.68% and 21.98% ± 0.38%, respectively (Figure 2A). In contrast, PCA/C MFCs showed negligible responses at these concentrations (0.79% ± 0.44% and 2.61% ± 0.48%, respectively). This demonstrates that sensors utilizing PCA/GAMP‐H biofilms achieve significantly lower Cd(II) detection limits, capable of detecting 0.03 mg L^−1^. Comparison of our biosensor results with those of other reported electrochemical biosensors is summarized in Table S3. The genetically engineered *G. sulfurreducens* biofilm biosensor achieves a markedly lower detection limit (0.03 mg·L^−1^) and faster response time (15 min) than existing MFC‐based Cd(II) sensors, while maintaining a comparable inhibition ratio. Although techniques such as chemically modified electrodes offer ultra‐low detection limits (Kokab et al. 2020), their reliance on sophisticated instrumentation limits their applicability for continuous, on‐site monitoring. In contrast, the self‐powered MFC platform presents an economical and sustainable alternative, ideally suited for continuous ecotoxicological surveillance. The achieved detection limit of 0.03 mg L^−1^ for Cd(II) is well suited for industrial wastewater monitoring, as it is three times lower than China's discharge standard of 0.1 mg L^−1^ (Ministry of Environmental Protection 1996), enabling effective early warning. However, it remains an order of magnitude above the World Health Organization (WHO) drinking water guideline (0.003 mg L^−1^) (World Health Organization 2022), and thus not yet applicable for potable water assessment. Further optimization is needed to meet stricter regulatory requirements.

Sensor sensitivities were further analysed using linear calibration curves. Data points for PCA/C MFCs at 0.03 and 0.06 mg L^−1^ Cd(II) were excluded from calibration due to negligible responses. As shown in Figure 2B,C, IRs from PCA/GAMP‐H biofilm‐based sensors exhibited superior linear correlation with Cd(II) concentrations (*R* ^2^ = 0.9823) compared to PCA/C sensors (*R* ^2^ = 0.7968). Sensitivity, defined as the electrical signal change per unit analyte concentration change (Di Lorenzo et al. 2014), was quantified by the slope of the fitted concentration‐response line. The sensitivity for Cd(II) detection was 12.60%/mg for PCA/GAMP‐H‐based sensors versus 8.88%/mg for PCA/C‐based sensors (Figure 2B,C). These results confirm that the engineered *G. sulfurreducens* biofilms significantly improve biosensor sensitivity for Cd(II) detection. To further assess the generalizability of the engineered cGAMP‐elevated biofilm for enhanced toxicant sensitivity, we extended our analysis beyond Cd(II) to include hexavalent chromium [Cr(VI)] as an additional model heavy metal (Figure S4A). Upon exposure to 1.5 mg L^−1^ Cr(VI), a concentration aligned with the Chinese discharge standard (Ministry of Environmental Protection 1996), MFC biosensors functionalized with the PCA/GAMP‐H biofilm exhibited a voltage inhibition ratio of 19.15% ± 5.72%, significantly higher than the 5.70% ± 1.68% observed for the PCA/C control biofilm under identical conditions (Figure S4B). These results indicate that the improved sensitivity conferred by elevated intracellular cGAMP is not restricted to Cd(II) but extends at least to Cr(VI), suggesting a broader enhancement of the electroactive biofilm's responsiveness to heavy metal stress.

Biosensor reusability is essential for toxicity detection, particularly when toxicant concentrations fluctuate between low and high levels. To address this, we performed three consecutive Cd(II) sensing cycles with stepwise exposure to 0.1, 0.6 and 1.2 mg/L Cd(II). After reaching steady state (100–120 h), PCA/GAMP‐H MFCs generated a voltage of 605.41 ± 34.92 mV, approximately 136 mV higher than that of PCA/C controls (468.94 ± 23.68 mV, *p* < 0.05). To assess the reusability of PCA/GAMP‐H biofilm‐based MFC biosensors for daily Cd(II) monitoring, a 40 h recovery period was introduced before the second and third sensing cycles. After the first recovery, PCA/GAMP‐H biofilms restored ~94% of their initial voltage and maintained a 23.5% higher output than PCA/C (574.15 ± 8.64 vs. 464.85 ± 10.67 mV, *p* < 0.05). Following the second recovery, the voltage gap between PCA/GAMP‐H and PCA/C narrowed (561.78 ± 18.28 vs. 444.61 ± 11.21 mV, *p* < 0.05), while the Cd(II) response of PCA/GAMP‐H remained stable across cycles (Figure 2D). Notably, Cd(II) exposure induced a more pronounced voltage drop in PCA/GAMP‐H biofilms than in PCA/C controls (Figure 2D). Moreover, the pYYDT vector in *G. sulfurreducens* PCA during MFC‐based biosensor operation is stable (Figure S5). Overall, PCA/GAMP‐H biofilms exhibited enhanced sensitivity to Cd(II) and robust reusability, sustaining higher voltage output and stable detection performance despite repeated toxic exposures.

### 2.3 The Improved Sensitivity of the MFC‐Based Biosensor Resulted From Increased *c*‐Cyt Expression and Reduced Exopolysaccharide Production

**FIGURE 3:**
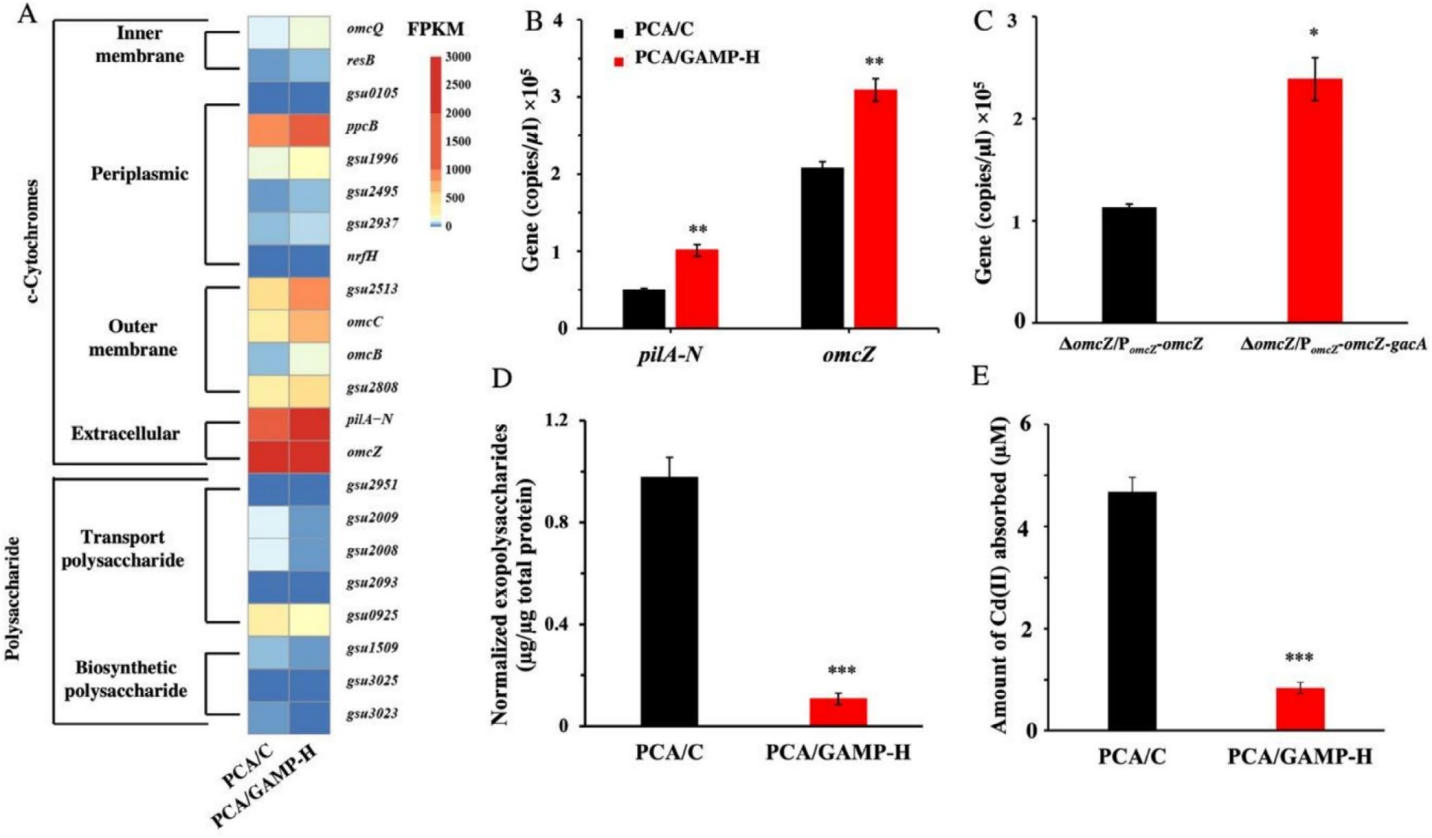
Enhanced sensitivity of MFC‐based sensing is attributed to upregulated *c*‐Cyts and nanowires, along with reduced exopolysaccharide production. (A) Expression levels of genes encoding *c*‐Cyts, nanowires and exopolysaccharide transport/biosynthesis in PCA/C and PCA/GAMP‐H cells. (B) qPCR analysis of nanowire‐associated gene expression in PCA/C and PCA/GAMP‐H anode biofilm cells. (C) qPCR analysis of *omcZ* transcription in Δ*omcZ*/P_*omcZ*_‐*omcZ* and Δ*omcZ*/P_*omcZ*_‐*omcZ*‐*gacA* strains with elevated cGAMP levels. (D) Polysaccharide content in the biofilm matrix normalized to total protein. (E) Cd(II) sequestration capacity of EPS extracted from PCA/C and PCA/GAMP‐H biofilms. Data are mean ± SD (*n* = 3 independent samples). Two‐sided Student's *t*‐test was used to analyse the statistical significance (*p < 0.05, **p < 0.01, ***p < 0.001).

To explore the mechanism underlying the enhanced EET efficiency and biosensor performance, we performed RNA‐seq transcriptomic analysis. Comparative expression profiling of the PCA/GAMP‐H strain versus the control PCA/C strain identified 372 differentially expressed genes, including 150 upregulated and 222 downregulated genes (Figure S6). Notably, genes encoding *c*‐Cyts critical for EET exhibited significantly elevated expression levels in PCA/GAMP‐H (Figure 3A and Table S4). The upregulated *c*‐Cyts localized across various subcellular compartments: the inner membrane, periplasm and outer membrane. This pattern of *c*‐Cyt upregulation aligns with our previous findings in mineral‐attached PCA/GAMP‐H biofilms (Xu et al. 2025). Furthermore, PCA/GAMP‐H showed increased transcription levels for both *omcZ* and *pilA* genes encoding extracellular conductive nanowires (Figure 3A). Their expression in biofilm cells was further confirmed by qPCR, which is consistent with transcriptomic analysis (Figure 3B). The primers used for qPCR are shown in Table S5. The established roles of OmcZ and PilA in facilitating electron transport across the *G. sulfurreducens* biofilm matrix underscore the functional significance of their upregulation. To establish a causal link between cGAMP and EET‐associated gene upregulation, we performed a genetic complementation of the *omcZ* deletion mutant. Compared to the control strain (Δ*omcZ*/P_*omcZ*_‐*omcZ*), the strain with elevated cGAMP (Δ*omcZ*/P_*omcZ*_‐*omcZ*‐*gacA*) showed restored upregulation of *omcZ* transcription (Figure 3C). Given that increased *omcZ* transcription is known to enhance EET (Hu et al. 2024), this demonstrates that cGAMP partly promotes EET by directly activating *omcZ* transcription. The coordinated overexpression of these EET‐related genes, spanning from the inner membrane through the periplasm to the outer membrane and extending into the biofilm matrix, likely constitutes an optimized electron conduction pathway from the cell interior to the anodes. This enhancement in the electron transfer system provides a plausible explanation for the low internal resistance and high EET efficiency observed in PCA/GAMP‐H biofilms.

Elevated intracellular cGAMP levels in PCA/GAMP‐H also significantly decreased mRNA levels of genes essential for exopolysaccharide production compared to PCA/C (Figure 3A and Table S6). Specifically, transcriptional downregulation was observed for both ABC transporter genes, which are critical for polysaccharide export, as well as glycosyltransferase genes involved in exopolysaccharide biosynthesis. This coordinated downregulation in anode biofilm cells was further confirmed using qPCR (Figure S7). Moreover, consistent with the observed transcriptional suppression, direct measurement of exopolysaccharide extracted from PCA/GAMP‐H anode biofilms revealed a 9‐fold reduction when normalized to biofilm biomass (0.11 ± 0.02 vs. 0.98 ± 0.08 μg polysaccharide/μg total protein in PCA/C; Figure 3D). These results suggest that cGAMP suppresses polysaccharide synthesis in extracellular polymeric substances (EPS) matrix. Given the role of exopolysaccharide in biofilm formation, this diminished polysaccharide production provides a mechanistic explanation for the thinner anode biofilms observed in PCA/GAMP‐H MFCs. The relatively low exopolysaccharide abundance in the PCA/GAMP‐H biofilm likely contributed to its lower Cd(II) sequestration capacity (Figure 3D,E). In EAB‐based sensors, toxicants primarily disrupt sensor output by inhibiting intracellular electron generation processes (Qi et al. 2023); therefore, efficient diffusion of toxicants through the biofilm to reach cells is critical for generating detectable signal perturbations. However, exopolysaccharides serve as a protective barrier by sequestering toxicants, especially heavy metals, thereby reducing their bioavailability to cells (Yi et al. 2019; Qi et al. 2021). Consequently, the diminished exopolysaccharides production in PCA/GAMP‐H likely enhances sensor sensitivity by minimizing this protective sequestration effect, thereby amplifying signal responses. This proposed mechanism is supported by the previous study that used different anode potentials to regulate EPS and their responses to toxic substances (Hou et al. 2020).

The interfaces between the solution‐biofilm and biofilm‐electrode are crucial for mass and electron transfer, significantly impacting the sensitivity of EAB‐based sensors (Qi et al. 2021). Notably, this study reveals that elevated intracellular levels of cGAMP enhance the performance of PCA/GAMP‐H biofilms as sensing elements, likely by facilitating electron transfer through the upregulation of EET proteins and reducing mass transfer barriers via decreased polysaccharide production. These combined effects, enhanced electron transfer and diminished diffusion resistance, substantially improve the sensitivity of EAB‐based sensors in detecting Cd(II). While reduced EPS is often associated with decreased biofilm stability, our MFC biosensor maintained stable performance over three Cd(II) exposure cycles despite lower EPS levels (Figure 3D). This suggests a nuanced trade‐off: enhanced sensitivity achieved by reducing Cd(II) sequestration does not necessarily lead to immediate loss of functional stability. We hypothesize that, within the applied stress regime, the biofilm's core architecture remained structurally and functionally intact, and that improved substrate mass transfer may have offset the diminished extracellular protective capacity. Moreover, beyond metal sequestration, EPS also protects biofilms from predation, shear stress and microbial competition (Flemming et al. 2016). Our study shows that reducing EPS enhances sensor sensitivity without compromising short‐term electrochemical stability under controlled conditions. However, this may carry ecological trade‐offs in complex environments, for example, increased vulnerability to protozoan grazing or displacement by EPS‐producing competitors (Flemming et al. 2025). Such risks are likely limited for MFC‐based biosensors in closed reactors. Moreover, translating this strategy to field applications faces challenges from environmental fluctuations and long‐term stability issues (e.g., biofilm dispersal), as well as scale‐up effects like increased internal resistance that may reduce sensitivity. Despite these hurdles, the self‐powered and continuous monitoring capabilities of MFCs remain attractive, and future efforts should assess long‐term performance of engineered biofilms under environmentally relevant conditions, including mixed communities and natural fluctuations.

## 3 Conclusions

EABs play a pivotal role in sustainable technologies for bioenergy production, environmental monitoring, and bioremediation. This study demonstrates that engineering *G. sulfurreducens* biofilms through elevated levels of cGAMP significantly improves the sensitivity of EAB‐based sensors for detecting Cd(II). Given that cadmium is a persistent and bioaccumulative toxin, the achieved 20‐fold reduction in the detection limit for Cd(II) facilitates earlier warnings of water contamination, an essential factor for protecting ecosystems and public health. Furthermore, the mechanistic insights reveal enhanced EET proteins coupled with reduced exopolysaccharide production in engineered biofilms, providing a universal framework for optimizing EAB‐based systems. Despite the enhanced sensitivity achieved through engineering biofilms, limited specificity remains a persistent challenge for EAB‐based biosensors (Hu et al. 2022). To overcome this fundamental bottleneck, future design strategies should shift from conventional single‐signal outputs toward the integration of molecularly engineered recognition modules. In this context, genetically engineered *G*. *sulfurreducens* offers a promising chassis for incorporating analyte‐responsive transcriptional regulatory networks. By coupling specific transcription factors and their cognate promoters to the electrogenic output of electroactive bacteria, a generic toxicity response can be transformed into a highly specific molecular recognition event. For example, promoters that are selectively activated by heavy metals, organic pollutants or oxidative stress can be rewired to control the expression of key electrogenic elements such as *c*‐Cyts. This configuration ensures that current generation occurs only upon target analyte recognition, thereby enabling specific and programmable biosensing (Golitsch et al. 2013; Webster et al. 2014). This genetic engineering strategy holds promise for advancing in situ biosensor deployment in pollutant tracking.

## Author Contributions

**Yongguang Jiang:** methodology, supervision. **Hongqing Yin:** supervision, project administration, resources. **Yutong Zhang:** methodology, data curation, investigation, software, writing – review and editing, validation, formal analysis. **Yiran Dong:** writing – review and editing. **Liang Shi:** investigation, funding acquisition. **Xi Han:** methodology, data curation, investigation, writing – review and editing, visualization, validation, formal analysis. **Yidan Hu:** writing – review and editing, writing – original draft, methodology, investigation, funding acquisition.

## Funding

The National Natural Science Foundation of China (NSFC42202340)and the Natural Science Foundation of Hubei Province (2021CFB214).

## Conflicts of Interest

The authors declare no conflicts of interest.

## Supporting information


**Table S1:** Strains and plasmids used in this study.
**Table S2:** Performance of MFCs with different *G. sufurreducens* strains containing different levels of intracellular cyclic GMP‐AMP (cGAMP).
**Table S3:** Performance comparison of electrochemical biosensors for Cd(II) detection.
**Table S4:** The upregulation of c‐cytochrome and nanowire genes in PCA/GAMP‐H cells.
**Table S5:** The primers used in this study.
**Table S6:** The downregulation of genes involved in polysaccharide in PCA/GAMP‐H cells.
**Figure S1:** Electrochemical impedance spectroscopy (EIS) spectra of microbial fuel cells (MFCs) with the cyclic GMP‐AMP (cGAMP) increased strain PCA/GAMP‐H and the control strain PCA/C.
**Figure S2:** Cyclic voltammetry (CV) curves of MFCs inoculated with PCA/GAMP‐H strain with a high cGAMP level and the control strain PCA/C (*n* = 3 independent samples).
**Figure S3:** The reduced voltages observed in the microbial fuel cells (MFCs) with PCA/C and PCA/GAMP‐H biofilms after 60 min of exposure to varying concentrations of Cd(II), specifically (A) 0.03, (B) 0.06, (C) 0.6, (D) 3, (E) 4.5, and (F) 6 mg L−1.
**Figure S4:** Effects of 1.5 mg L−1 Cr(VI) exposure on MFCs inoculated with PCA/C and PCA/GAMP‐H biofilms. (A) Voltage drop recorded after 60 min of exposure. (B) Inhibition rates (IRs) calculated based on the voltage changes. Data are mean ± SD (*n* = 3 independent samples). Two‐sided Student's *t*‐test was used to analyse the statistical significance (*p < 0.05).
**Figure S5:** Stability of the pYYDT vector in *G. sulfurreducens* PCA during MFC‐based biosensor operation. (A) Agarose gel electrophoresis of plasmids extracted from planktonic and biofilm cells after the 11‐day biosensor stability experiment in the presence of antibiotics. The persistent presence of pYYDT and its derivatives throughout the experiment confirms vector maintenance. (B) Plasmid copy number determined by qPCR targeting the plasmid‐borne lacI gene and the chromosome‐encoded omcS gene. Copy number was calculated as the ratio of lacI to omcS gene copies. Data are mean ± SD (*n* = 3 independent samples). Two‐sided Student's *t*‐test was used to analyse the statistical significance (ns: no significance).
**Figure S6:** Volcano plots for differential gene expression analysis between PCA/GAMP‐H and the control strain PCA/C.
**Figure S7:** Gene expression analysis of genes associated with polysaccharide production in PCA/GAMP‐H and PCA/C anode biofilm cells, conducted via qPCR during peak voltage conditions in MFCs. Data are mean ± SD (*n* = 3 independent samples). Two‐sided Student's *t*‐test was used to analyse the statistical significance (*p < 0.05, **p < 0.01, ***p < 0.001).

## Data Availability

The data that support the findings of this study are available from the corresponding author upon reasonable request.
