# Supplementary material for: Genetically Boosting Electron Transfer in Electroactive Biofilms for Improved Sensitivity of Microbial Fuel Cell‐Based Biosensing
Source: Microb Biotechnol. 2026 Apr 21;19(4):e70356. doi: 10.1111/1751-7915.70356 (PMC13099583; doi:10.1111/1751-7915.70356)
Supplement: Supplementary file 1 — Table S1: Strains and plasmids used in this study. Table S2: Performance of MFCs with different G. sufurreducens strains containing different levels of intracellular cyclic GMP‐AMP (cGAMP). Table S3: Performance comparison of electrochemical biosensors for Cd(II) detection. Table S4: The upregulation of c‐cytochrome and nanowire genes in PCA/GAMP‐H cells. Table S5: The primers used in this study. Table S6: The downregulation of genes involved in polysaccharide in PCA/GAMP‐H cells. Figure S1: Electrochemical impedance spectroscopy (EIS) spectra of microbial fuel cells (MFCs) with the cyclic GMP‐AMP (cGAMP) increased strain PCA/GAMP‐H and the control strain PCA/C. Figure S2: Cyclic voltammetry (CV) curves of MFCs inoculated with PCA/GAMP‐H strain with a high cGAMP level and the control strain PCA/C (n = 3 independent samples). Figure S3: The reduced voltages observed in the microbial fuel cells (MFCs) with PCA/C and PCA/GAMP‐H biofilms after 60 min of exposure to varying concentrations of Cd(II), specifically (A) 0.03, (B) 0.06, (C) 0.6, (D) 3, (E) 4.5, and (F) 6 mg L−1. Figure S4: Effects of 1.5 mg L−1 Cr(VI) exposure on MFCs inoculated with PCA/C and PCA/GAMP‐H biofilms. (A) Voltage drop recorded after 60 min of exposure. (B) Inhibition rates (IRs) calculated based on the voltage changes. Data are mean ± SD (n = 3 independent samples). Two‐sided Student's t‐test was used to analyse the statistical significance (*p < 0.05). Figure S5: Stability of the pYYDT vector in G. sulfurreducens PCA during MFC‐based biosensor operation. (A) Agarose gel electrophoresis of plasmids extracted from planktonic and biofilm cells after the 11‐day biosensor stability experiment in the presence of antibiotics. The persistent presence of pYYDT and its derivatives throughout the experiment confirms vector maintenance. (B) Plasmid copy number determined by qPCR targeting the plasmid‐borne lacI gene and the chromosome‐encoded omcS gene. Copy number was calculated as the ratio of lacI to omc [file MBT2-19-e70356-s001.docx]

**Supplementary Information for**

**Genetically Boosting Electron Transfer in Electroactive Biofilms for Improved Sensitivity of Microbial Fuel Cell-Based Biosensing**

Yutong Zhang^a#^, Xi Han^a,b#^, Yongguang Jiang^a^, Yiran Dong ^a, c^, Liang Shi^a, c^, Hongqing Yin^d^, Yidan Hu^a*^

^a^ Department of Biological Sciences and Technology, School of Environmental Studies, China University of Geosciences, Wuhan 430074, China

^b^ The Key Laboratory of Water and Sediment Sciences, College of Environmental Sciences and Engineering, Peking University, Beijing 100871, China

^c^ State Key Laboratory of Biogeology and Environmental Geology, China University of Geosciences, Wuhan 430074, China

^d^ Enshi Tujia and Miao Autonomous Prefecture Academy of Agricultural Sciences, Enshi 445099, China

# These authors contributed equally.

*Correspondence

Corresponding Authors: huyidan@cug.edu.cn

The supplementary information consists of 23 pages, 6 tables and 7 figures.

**Supplementary note 1. Experimental Procedures**

**Bacterial strains and culture conditions.** Table S1 lists the strains and plasmids used in this study. The wild-type (WT) strain *Geobacter sulfurreducens* PCA was obtained from the American Type Culture Collection (ATCC). As described in our previous study (Hu, et al., 2024, Xu, et al., 2025), the engineered strain PCA/GAMP-H has increased intracellular cGAMP levels due to the expression of the cGAMP synthase gene *gacA*. A control strain, PCA/C, carrying an empty vector, was also generated. All bacterial strains were cultivated anaerobically at 30°C in NBAF medium with 20 mM acetate as the electron donor and 40 mM fumarate as the electron acceptor. Kanamycin (200 μg mL^-1^) was added to the growth medium when necessary.

**Plasmid stability.** In our previous studies (Hu, et al., 2024, Xu, et al., 2025)., the stability of the pYYDT vector in Geobacter sulfurreducens PCA was enhanced through a plasmid-host adaptation process. To evaluate vector stability in the present work, plasmids were extracted from both planktonic and biofilm cells of G. sulfurreducens following an 11-day microbial fuel cells (MFC)-based biosensor stability experiment conducted in the presence of antibiotics. The presence of pYYDT and its derivatives in the host cells was monitored throughout the experimental period by plasmid extraction and subsequent gel electrophoresis analysis (Fig. S5A). This routine monitoring confirmed that the plasmids were consistently maintained over the course of the experiment.

The copy number of the pYYDT vector was determined using previously established quantitative polymerase chain reaction (qPCR) -based methods (Lee, et al., 2006). Specifically, qPCR was performed using primer sets targeting the plasmid-borne lacI gene and the chromosome-encoded omcS gene. As both lacI and omcS are present as single-copy sequences on the plasmid and chromosome, respectively, the plasmid copy number was calculated as the ratio of lacI to omcS gene copies. The average copy number of pYYDT was found to be 31 ± 2 prior to the biosensor stability test and 29 ± 2 after 11 days, with no statistically significant difference (ns, *p* > 0.05) (Fig. S5B). These results indicate that the pYYDT vector remained relatively stable in G. sulfurreducens PCA throughout the duration of the experiment.

**Bioelectronic device setup and electrochemistry characterization.** As previously established (Hu, et al., 2024), two-chamber MFCs were set up using a Nafion 117 membrane for separation. The anode (1 cm × 1 cm) and cathode (2.5 cm × 3 cm) electrodes were made of carbon cloth (WOS1002). The cathode comprised 50 mM K₃[Fe(CN)₆] and phosphate buffer, while the anode contained 150 mL of fumarate-free NBFA medium with electrodes as electron acceptors. Cultures were added to the anode at OD600 = 0.4 and incubated at 30°C. Voltage was recorded across a 2 kΩ resistor, with triplicate experiments conducted. At peak voltage, polarization curves and electrochemical impedance spectroscopy (EIS) were measured using a CHI 1000C workstation with an Ag/AgCl reference electrode. Moreover, the maximum electron flux through biofilms and individual cells was estimated using previous methods (Hu, et al., 2024).

**Images and biomass measurement of anode biofilm.** The biofilms on the anodes were stained with 2.5 μM SYTO 9, a fluorescent nucleic acid dye, and visualized using a confocal laser scanning microscope (CLSM, Leica Microsystems CMS GmbH) (Hu, et al., 2024). Fluorescence imaging was performed at excitation wavelengths of 488 nm and 561 nm, with image analysis conducted using Leica LAS X software. To further quantify the biomass on the electrodes, carbon cloth samples were cut into smaller sections and immersed in 0.22 mM NaOH solution. The cells were lysed by heating at 96°C for one hour while shaking gently to ensure complete disruption of the biofilm cells. After lysis, a 5 µL sample of the supernatant was then analyzed for total protein content using the Qubit protein assay kit and a Qubit fluorometer (Thermo Fisher Scientific, MA, USA).

**Biosensor performance analysis.** The performance of the biosensor was assessed based on its sensitivity to Cd(II). To illustrate the sensitivity of the MFC-based biosensor utilizing PCA/C and PCA/GAMP-H biofilms as sensing elements, cadmium chloride (CdCl_2_) at varying final concentrations ranging from 0 to 6 mg L^-1^ was introduced once each MFC achieved a stable voltage output. The inhibition ratio (IR), employed for evaluating the sensing performance, was calculated using the following equation (Yi, et al., 2019, Qi, et al., 2021)：

IR(%) ＝ (V_0_－V_F_ )/V_0_ × 100%

where V_0_ represents the voltage of the MFC prior to toxicity exposure, and V_F_ denotes the minimum voltage recorded during the sensing period.

**RNA sequencing and data analysis.** Total RNA of PCA/C and PCA/GAMP-H cells grown in NBAF medium was extracted using the HiPure Universal RNA Mini Kit (Magen, Guangzhou, China) and evaluated for quality via electrophoresis on 1% agarose gels. RNA concentration was measured with a Qubit 3.0 fluorometer (Thermo Fisher Scientific, MA, USA). Further details on gene expression analysis are available in our previous studies (Hu, et al., 2024, Xu, et al., 2025). Briefly, gene expression analysis commenced with cluster generation on a cBot system, followed by sequencing of the prepared library on an Illumina NovaSeq 6000 platform (Magigene, China). Each sample was represented by three biological replicates for RNA‑seq. The raw sequencing data, which were part of our previous study but not analyzed therein (Xu, et al., 2025), have been submitted to the NCBI database under accession number PRJNA1143950. Gene‑level read counts and functional annotations were extracted from the alignments using HTSeq‑count. To enable cross‑gene and cross‑experiment comparisons, expression levels were normalized to fragments per kilobase of transcript per million mapped reads (FPKM). Differential expression between two groups was assessed with edgeR, and the resulting *p*‑values underwent Benjamini–Hochberg correction to control the false discovery rate. Genes were considered significantly differentially expressed if they met the criteria of *p* < 0.05. Differentially expressed genes related to *c*-Cyts and exopolysaccharide production in anode biofilm cells were further validated by qPCR. A list of primers used is provided in Table S5. qPCR data were generated from three biological replicates.

**Exopolysaccharide extraction and determination.** After the voltage outputs of the MFCs peaked, exopolysaccharides from the extracellular polymeric substances (EPS) of biofilms on the electrodes were extracted using the EDTA method as previously described (Cao, et al., 2011). The concentrations of the extracted exopolysaccharides were determined using the phenol-sulfuric acid method (Zhuang, et al., 2020). Briefly, 1 mL of the extract was placed in a centrifuge tube, followed by an equal volume of 6% phenol and 5 mL of concentrated sulfuric acid (98%). After thorough mixing, color development occurred at room temperature for 30 minutes. Absorbance was then measured at 490 nm with a microplate reader (Thermo Fisher Scientific, USA).

**Cd(II) adsorption experiments**. Exopolysaccharides were extracted from PCA/C and PCA/GAMP-H anode biofilms using the EDTA method as described above (Cao et al., 2011). The exopolysaccharides solution was mixed with three volumes of ethanol, precipitated at −20 °C for 48 h, and centrifuged to collect the pellet. The precipitate was redissolved in ultrapure water, dialyzed (12 kDa membrane) against water for 24 h, and lyophilized (ALPHA1-4 LD plus, Christ, Germany) to obtain dry EPS.

For adsorption assays, the exopolysaccharides powder was introduced into 5 mL of NBFA medium containing 1.2 mg/L Cd(Ⅱ). The mixtures were incubated at 30 °C for 24 h and then filtered through 0.2 μm membranes. Residual Cd(Ⅱ) was quantified by inductively coupled plasma mass spectrometry (ICP‑MS, AvioTM 500, PerkinElmer, USA).

**Biosensor stability experiments.** Once the voltage increase rates of both PCA/C and PCA/GAMP-H MFCs became stabilized, Cd(II) sensing was initiated. Following a 2-hour exposure to Cd(II), the anode chamber was drained and refilled with fresh anolyte inside an anaerobic chamber. Concurrently, the catholyte was replaced to maintain stable cathode performance. The refreshed MFCs were then allowed to recover before subsequent Cd(II) sensing cycles. To evaluate reusability, prior to the second and third sensing cycles, both PCA/C and PCA/GAMP-H biofilms were allowed to recover in Cd(II)-free medium to restore comparable voltage. Each MFC was subjected to three sequential Cd(II) sensing cycles within a single experiment. The tested Cd(II) concentrations were selected based on the industrial wastewater discharge limit (0.1 mg L⁻¹) specified in National Standard GB 8978-1996 by the Ministry of Ecology and Environment. To assess sensing performance under fluctuating toxicant levels, the MFCs were sequentially exposed to Cd(II) at concentrations of 0.1, 0.6, and 1.2 mg/L. All experiments were performed in triplicates.

**Quantification of biofilm conductivity.** The conductivity of biofilms was assessed using a commercially available interdigitated array (IDA) of gold microelectrodes, following a previously established protocol (Yates et al, 2018). A three-electrode configuration was employed, with the IDA serving as the working electrode, an Ag/AgCl reference electrode, and a platinum wire counter electrode. Bacterial suspensions with an optical density at OD₆₀₀ of 0.5 were inoculated into the culture medium and cultivated at 30°C under a constant potential of 0 V (vs. Ag/AgCl) until biofilm maturation. The apparatus was then transferred into an anaerobic glove box, and the electrolyte was replaced with a solution free of sodium fumarate and sodium acetate. After 1 h of equilibration, electrochemical gating (E_G_) was applied to characterize the biofilm conductivity.

Linear sweep voltammetry (LSV) was performed using a bipotentiostat (IGS1200, Ingsens, China)over a potential range of –0.7 V to +0.3 V (vs. Ag/AgCl) at a scan rate of 0.001 V s⁻¹, with a fixed source–drain voltage (V_SD_) of 0.01 V, yielding the source–drain current (I_SD_) as a function of the gating potential. After the electrical measurements, the IDA electrodes were retrieved, and biofilm thickness was determined by CLSM (TCS SP8, Leica, Germany). The conductivity (σ) of the biofilm was calculated according to the following geometric relationships:

I_SD_ = G · V_SD_
G = σ · S

where G is the conductance and S is a system‑dependent geometric scaling factor that incorporates electrode dimensions, inter‑electrode gap width, and biofilm height. The factor S can be evaluated numerically using finite‑element modeling software, as described in previous studies (Yates, et al, 2015).

**Statistics and reproducibility.** GraphPad Prism (version 9.0.0) was employed for all statistical analyses. Experiments were conducted in at least triplicate, and data are shown as mean ± standard deviation. To compare groups, a two-tailed Student’s *t*-test was used. Significance levels are indicated by asterisks: * *p* < 0.05, ** *p* < 0.01, *** *p* < 0.001; ns denotes not significant (*p* > 0.05).

**Supplementary Note 2. Estimation of the maximum electron flux through biofilms and individual cells**

The number of cells colonized on the electrode was estimated using a previously reported method (Yu, et al., 2020). As demonstrated in our previous study, a single *Geobacter sulfurreducens* PCA cell contains approximately 6.93 × 10^-7^ µg of protein. To determine the protein content of the biofilm on the electrode, the electrode was cut into pieces and mixed with 0.1 mM NaOH at 96°C for one hour to lyse the cells. Following centrifugation, the protein content in the supernatant was quantified using the Qubit Protein Assay Kit (version 3). The total protein concentrations and corresponding cell numbers for PCA/C and PCA/GAMP-H are presented as follows:

| Strain | Total proteins (μg) | Cell numbers |
| --- | --- | --- |
| PCA/C | 354.9 | 5.12×10^8^ |
| PCA/GAMP-H | 252.0 | 3.63×10^8^ |

The maximum electron flux through anode biofilms and individual cells were calculated using the following equation (Yu, et al., 2020)：

Max. e_f_ flux through biofilms＝I_max_ × t × (6.24×10^18^)

where I_max_ is the maximum output current when polarization curves were measured, t is the time defined as 1 second, and 6.24 × 10^18^ is the number of electrons when the quantity of charge (electricity) in coulombs is 1.

**Table S1.** Strains and plasmids used in this study.

| **Strain or plasmid** | **Description** | **Source** |
| --- | --- | --- |
| *Geobacter sulfurreducen* PCA | |  |
| *G. sulfurreducen* PCA | Wild type (WT) | Lab stock |
| PCA/GAMP-H | WT *G. sulfurreducen* carrying cGAMP synthase-encoding plasmid pGacA | (Xu, et al., 2025) |
| PCA/C | WT *G. sulfurreducen* carrying the empty vector pYYDT | (Hu, et al., 2024) |
| **Plasmids** |  |  |
| pYYDT | KmR; *oriV*(pBBR1), P*tac* | (Yang, et al., 2015) |
| pGacA | Overexpress *Gsu1658* gene | (Xu, et al., 2025) |

| Strain | P_max_ (W m^-2^) | Max.e_f_ flux through biofilms (electrons s^-1^)^*^ | Max.e_f_ flux  (electrons s^-1^ cell^-1^)^*^ |
| --- | --- | --- | --- |
| PCA/C | 0.51±0.03 | (0.99±0.12)×10^15^ | (1.93±0.23)×10^6^ |
| PCA/GAMP-H | 1.22±0.04 | (2.48±0.17)×10^15^ | (6.81±0.33)×10^6^ |

**Table S2.** Performance of MFCs with different *G. sufurreducens* strains containing different levels of intracellular cyclic GMP-AMP (cGAMP).

^*^The maximum electron flux per cell was estimated from the maximum current output reached in polarization curves.

**Table S3.** Performance comparison of electrochemical biosensors for Cd(II) detection.

| **Sensing elements** | **Detection limits** | **Detection**  **time** | **Inhibition ratio** | **Reference** |
| --- | --- | --- | --- | --- |
| Metal–organic framework and conducting polymer | 0.3 mg L^-1^ | - | - | (Wang, et al., 2017) |
| Tripeptide derivative-modified glassy carbon electrode | 4.88×10^−10^ mg L^-1^ | - | - | (kokab, et al., 2020) |
| *E. coli* cells with electron mediators | 2 mg L^-1^ | 120 mins | - | (Yang, et al., 2018) |
| Immobilized Saccharomyces cerevisiae cells | 5 mg L^-1^ | - | ~25% | (Gao, et al., 2017) |
| Anaerobic sludge | 2 mg L^-1^ | - | 9.29% | (Yu, et al., 2017) |
| Suspended *Shewanella oneidensis* | 0.1 mg L^-1^ | 30 mins | 16.7% | (Zang, et al., 2021) |
| Genetically engineered *G. sulfurreducens* biofilms | 0.03 mg L^-1^ | 15 mins | 11.2% | This study |

**Table S4.** The upregulation of *c*-cytochrome and nanowire genes in PCA/GAMP-H cells.
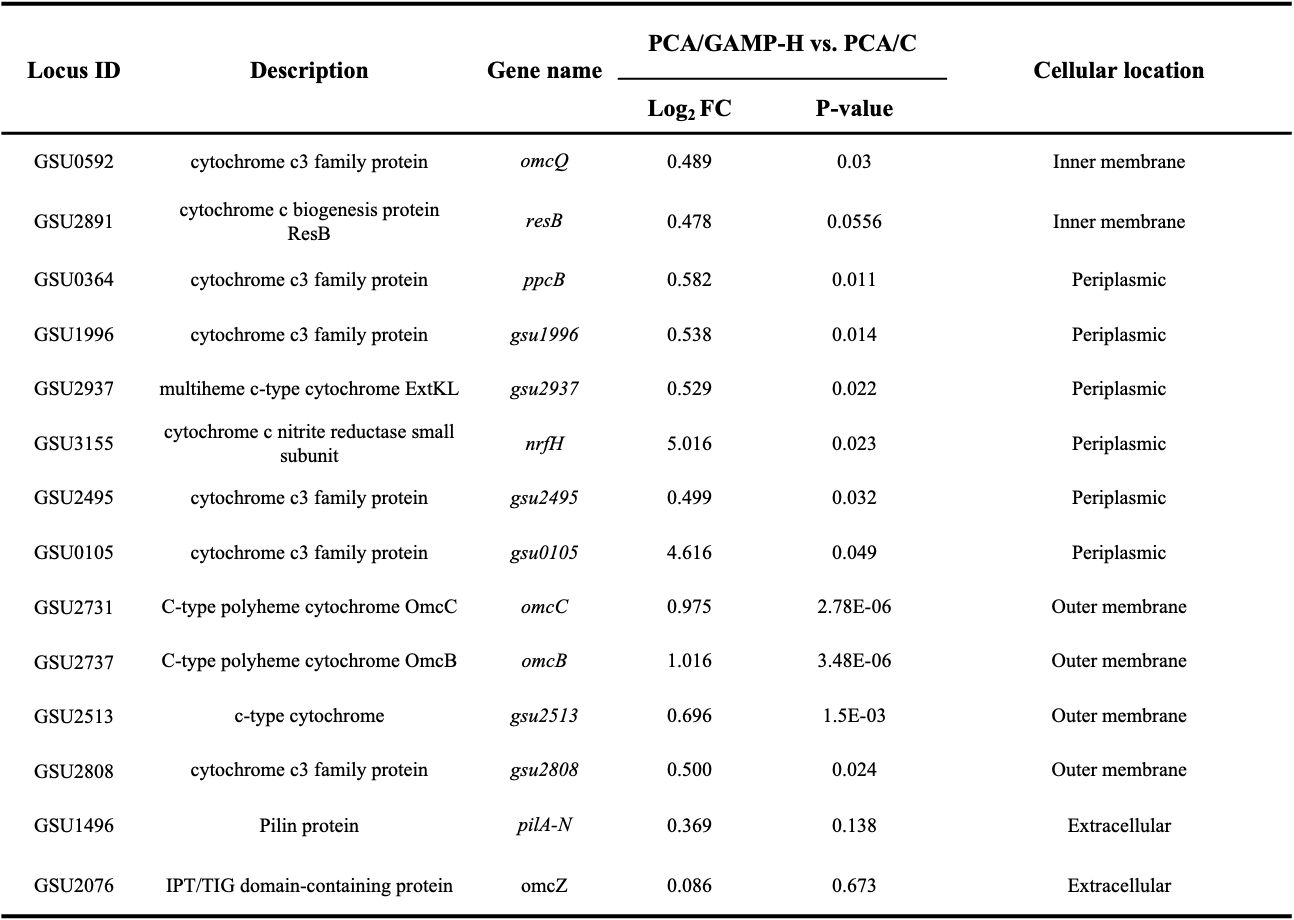


**Table S5.** The primers used in this study.

| Primer | Sequence (5’to 3’) | Description |
| --- | --- | --- |
| qpilA-F | actgctcttgagtccgcatt | qPCR quantification |
| qpilA-R | attgacagttcccgcggt | qPCR quantification |
| qomcE-F | gacgaacaccgaccagatctg | qPCR quantification |
| qomcE-R | cgtcatgacagctcatgcagaag | qPCR quantification |
| qomcZ-F | tttacggtcacgtagccgttg | qPCR quantification |
| qomcZ-R | ggcaagacccttaccattaccg | qPCR quantification |
| qomcS-F | cgtaggcaacgcgggtct | qPCR quantification |
| qomcS-R | atccccacgggaagtatcg | qPCR quantification |
| q0925-F | gcgctcttcgattctctcacg | qPCR quantification |
| q0925-R | gcatcaggtgctggatgttgtag | qPCR quantification |
| q1509-F | acactcgacctgatattcagttgc | qPCR quantification |
| q1509-R | cttgcaaccgagtactcgagc | qPCR quantification |
| q1759-F | cgaatctccaggcgatcatcg | qPCR quantification |
| q1759-R | gtgggccctgtgatcgatatg | qPCR quantification |
| q2008-F | gctaactcggacgtctccttc | qPCR quantification |
| q2008-R | accatgacgttgtcgagcac | qPCR quantification |
| q2009-F | tatcgacctggaggtcaagc | qPCR quantification |
| q2009-R | ccgcgggtactcttgagatag | qPCR quantification |
| q2093-F | tcctgcacggtctcgaatacg | qPCR quantification |
| q2093-R | aaatcagtcttcagcggcg | qPCR quantification |
| q3023-F | cgacttcatgtcaaactgcctgg | qPCR quantification |
| q3023-R | acacctccttgcgttaagtcg | qPCR quantification |
| q3025-F | gtcctcaacaagcaggacatcg | qPCR quantification |
| q3025-R | tgtcgttgaaccagctgaagatg | qPCR quantification |

**Table S6.** The downregulation of genes involved in polysaccharide in PCA/GAMP-H cells.


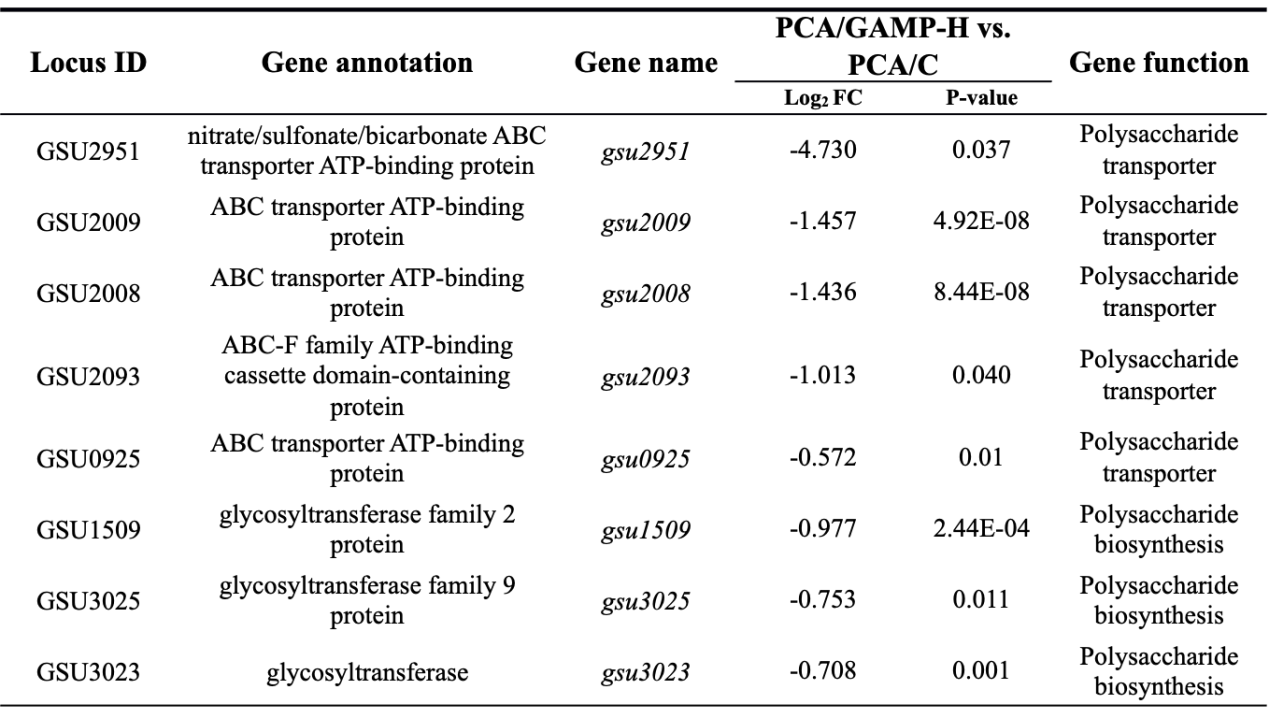


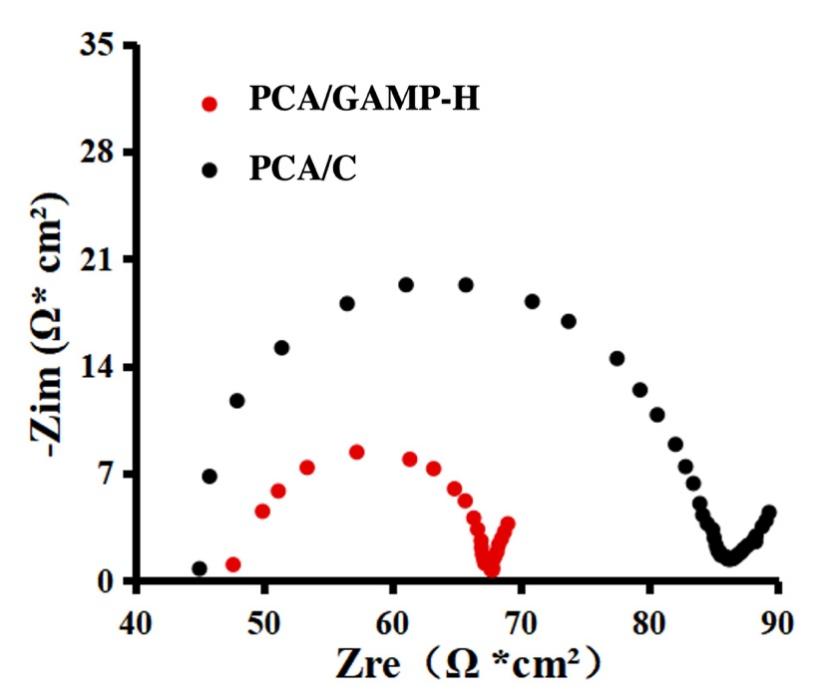


**Fig. S1** Electrochemical impedance spectroscopy (EIS) spectra of microbial fuel cells (MFCs) with the cyclic GMP-AMP (cGAMP) increased strain PCA/GAMP-H and the control strain PCA/C.


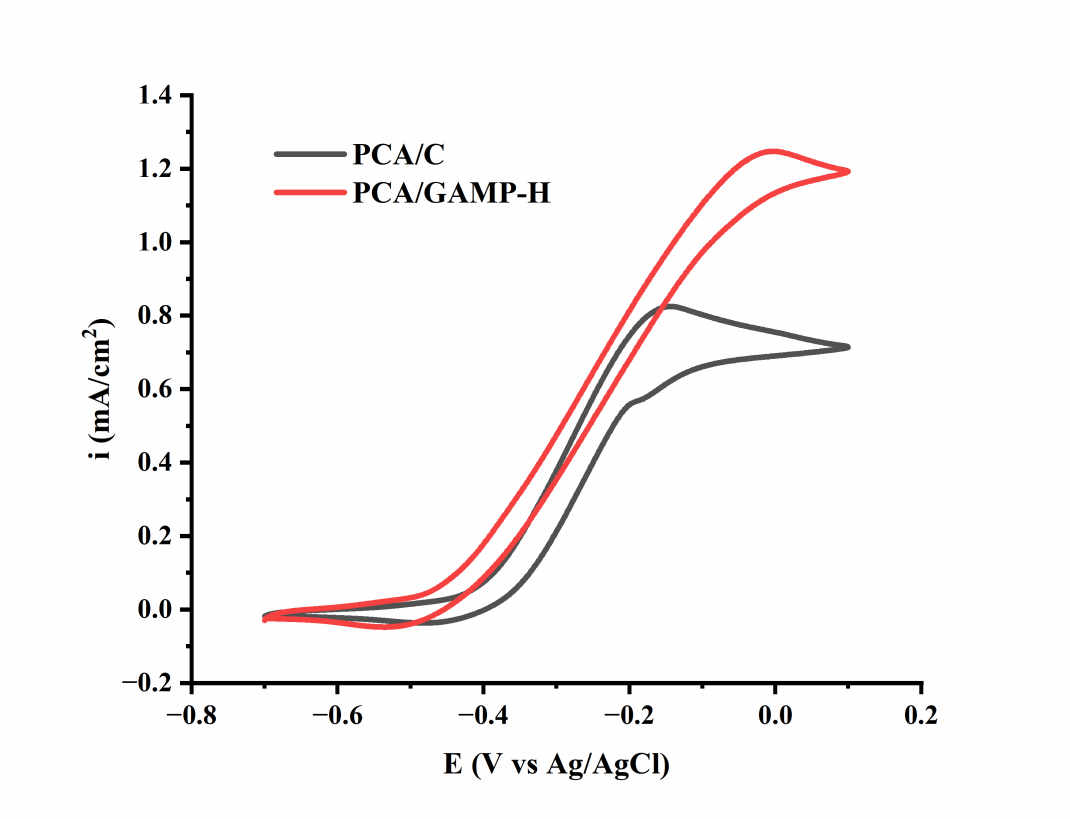


**Fig. S2** Cyclic voltammetry (CV) curves of MFCs inoculated with PCA/GAMP-H strain with a high cGAMP level and the control strain PCA/C (n = 3 independent samples).


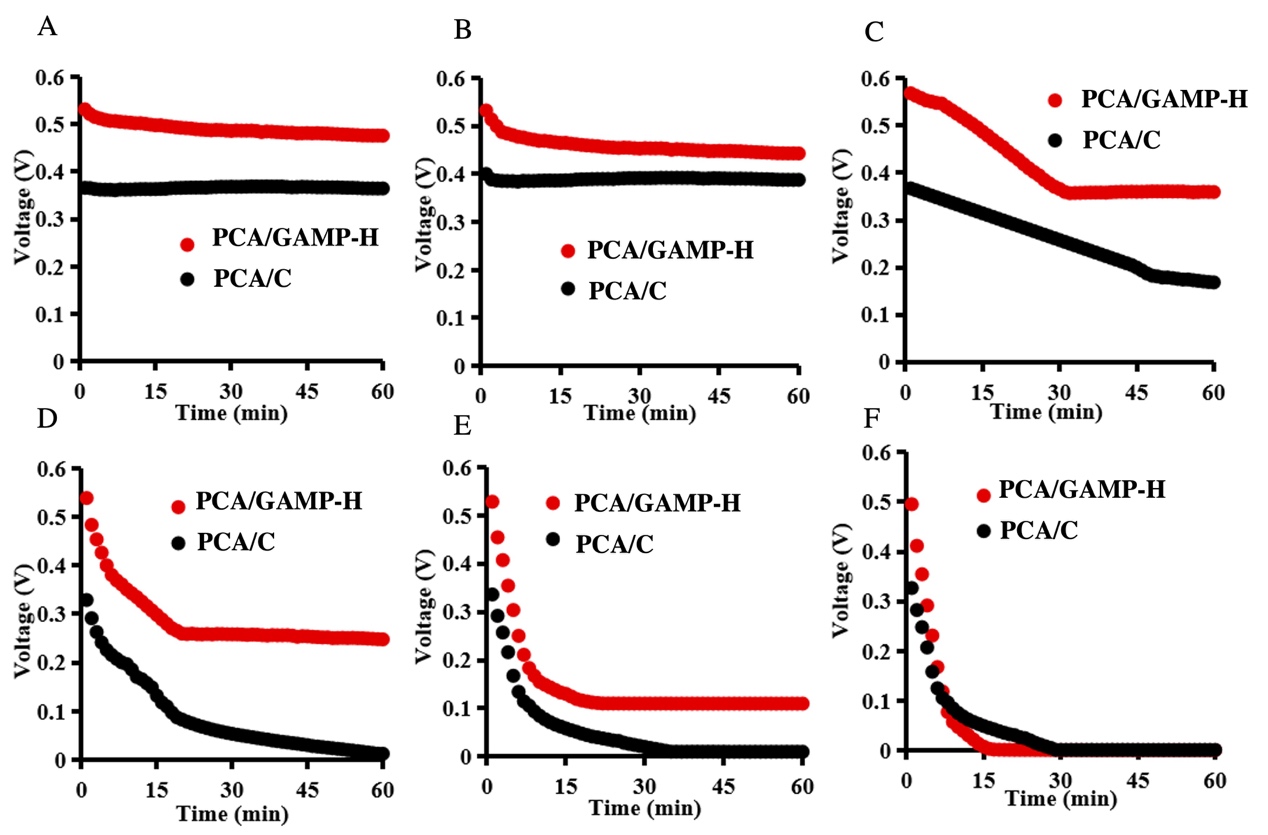


**Fig. S3** The reduced voltages observed in the microbial fuel cells (MFCs) with PCA/C and PCA/GAMP-H biofilms after 60 minutes of exposure to varying concentrations of Cd(II), specifically (A) 0.03, (B) 0.06, (C) 0.6, (D) 3, (E) 4.5, and (F) 6 mg L⁻¹.


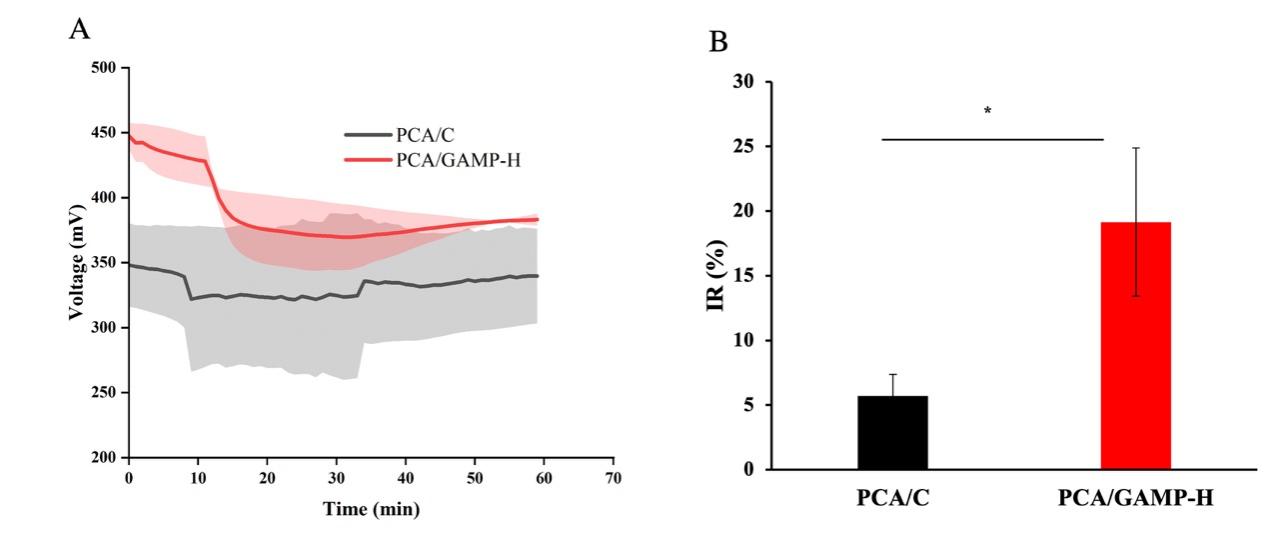


**Fig. S4** Effects of 1.5 mg L⁻¹ Cr(VI) exposure on MFCs inoculated with PCA/C and PCA/GAMP-H biofilms. (A) Voltage drop recorded after 60 minutes of exposure. (B) Inhibition rates (IRs) calculated based on the voltage changes. Data are mean ± SD (n = 3 independent samples). Two-sided Student’s *t* test was used to analyze the statistical significance (* *p* < 0.05).


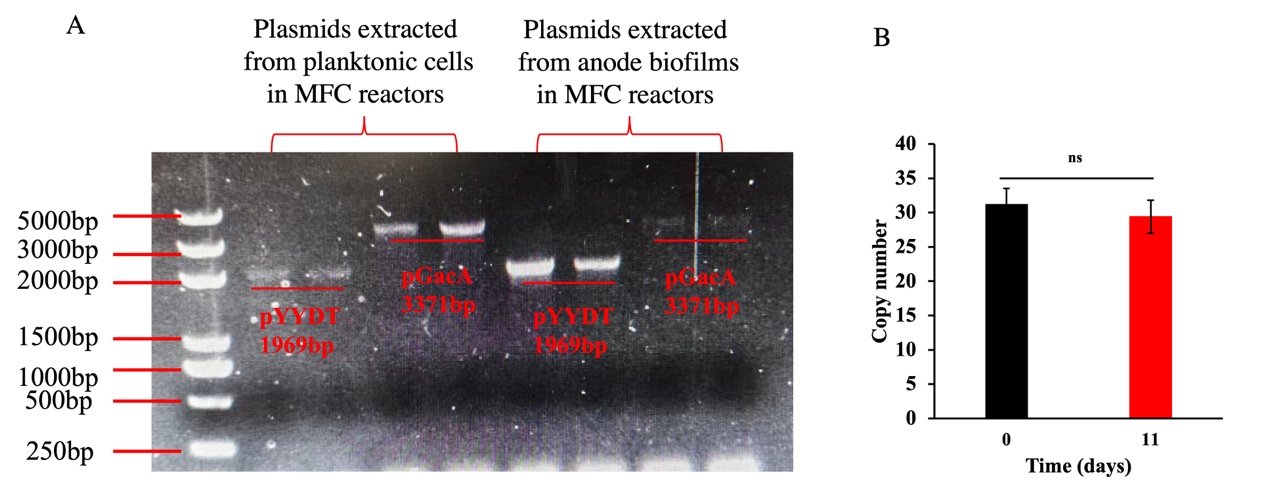


**Fig. S5** Stability of the pYYDT vector in *G. sulfurreducens* PCA during MFC-based biosensor operation. (A) Agarose gel electrophoresis of plasmids extracted from planktonic and biofilm cells after the 11-day biosensor stability experiment in the presence of antibiotics. The persistent presence of pYYDT and its derivatives throughout the experiment confirms vector maintenance. (B) Plasmid copy number determined by qPCR targeting the plasmid-borne *lacI* gene and the chromosome-encoded *omcS* gene. Copy number was calculated as the ratio of *lacI* to *omcS* gene copies. Data are mean ± SD (n = 3 independent samples). Two-sided Student’s *t* test was used to analyze the statistical significance (ns: no significance).


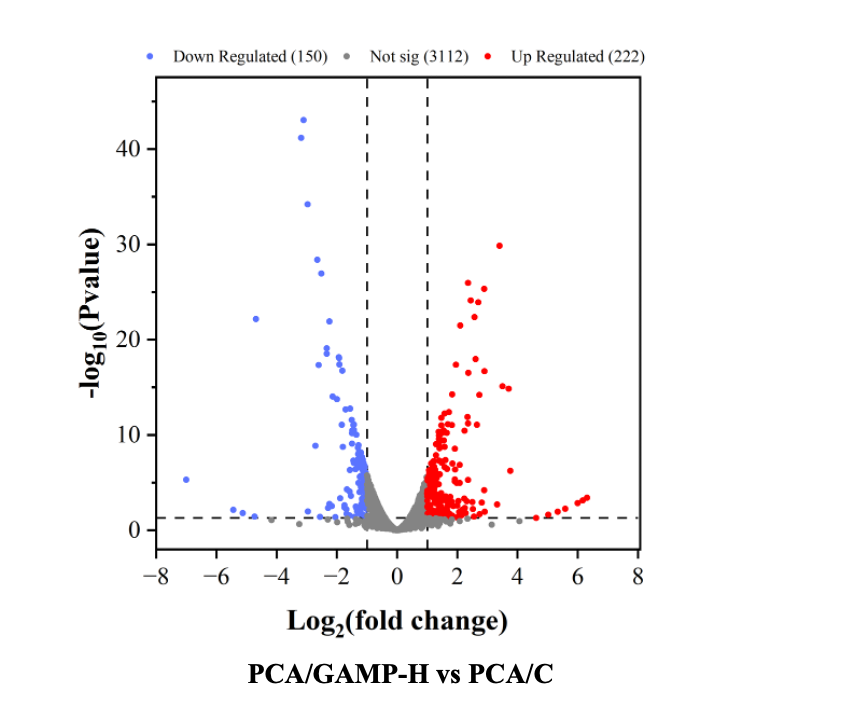


**Fig. S6** Volcano plots for differential gene expression analysis between PCA/GAMP-H and the control strain PCA/C.


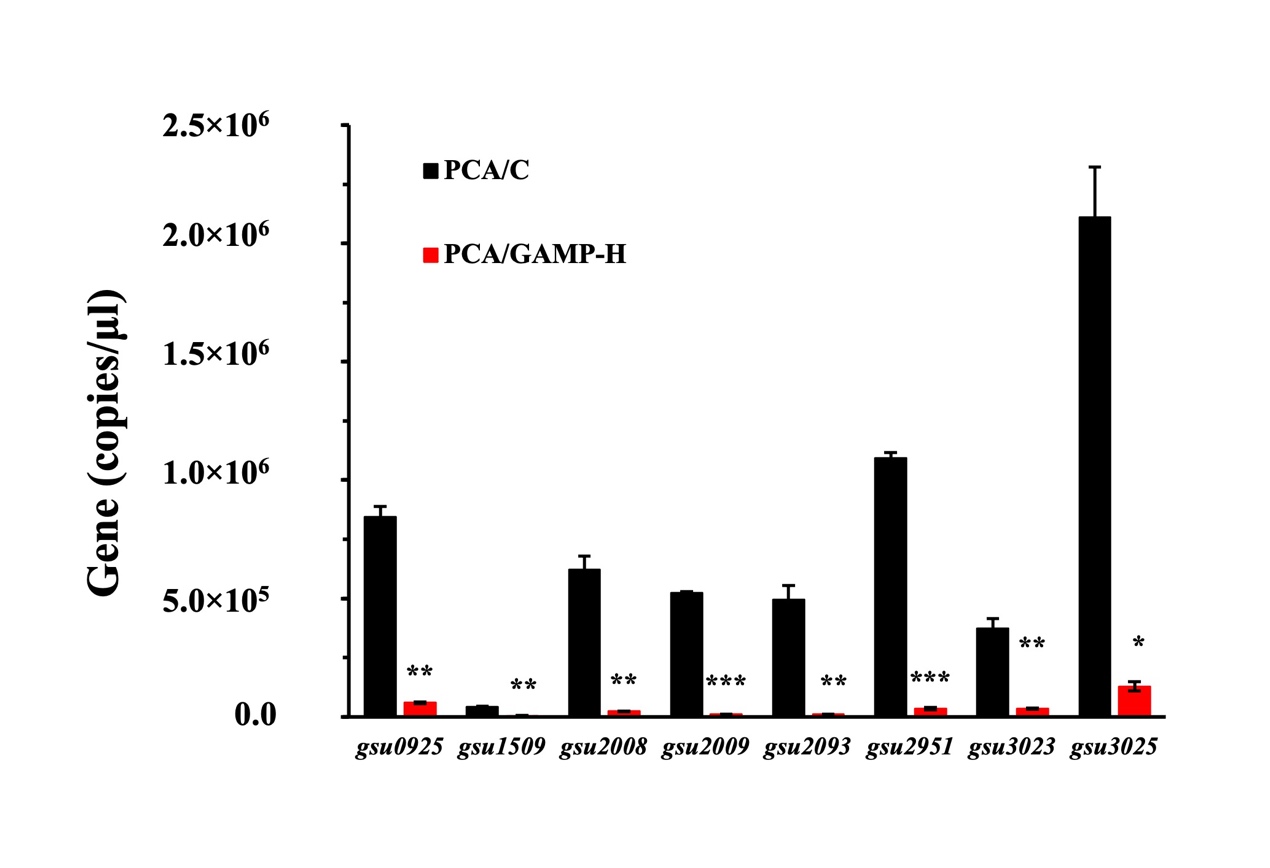


**Fig. S7** Gene expression analysis of genes associated with polysaccharide production in PCA/GAMP-H and PCA/C anode biofilm cells, conducted via qPCR during peak voltage conditions in MFCs. Data are mean ± SD (n = 3 independent samples). Two-sided Student’s *t* test was used to analyze the statistical significance (* *p* < 0.05, ** *p* < 0.01, *** *p* < 0.001).

**References：**

Cao, B., Shi, L., Brown, R.N., Xiong, Y., Fredrickson, J.K., Romine, M.F., et al. (2011) Extracellular polymeric substances from Shewanella sp. HRCR-1 biofilms: characterization by infrared spectroscopy and proteomics, *Environmental Microbiology* **13**: 1018-1031.

Gao, G., Fang, D., Yu, Y., Wu, L., Wang, Y., and Zhi, J. (2017) A double-mediator based whole cell electrochemical biosensor for acute biotoxicity assessment of wastewater, *Talanta* **167**: 208-216.

Hu, Y., Han, X., Luo, Y., Jiang, J., Jiang, Y., Cao, B., et al. (2024) All roads lead to Rome: Cyclic di-GMP differentially regulates extracellular electron transfer in *Geobacter* biofilms, *The Innovation Life* **2**: 100052.

kokab, T., Shah, A., Nisar, J., Khan, A.M., Khan, S.B., and Shah, A.H. (2020) Tripeptide derivative-modified glassy carbon electrode: a novel electrochemical sensor for sensitive and selective detection of Cd^2+^ ions, *ACS Omega* **5**: 10123-10132.

Lee, C., Kim, J., Shin, S.G., and Hwang, S. (2006) Absolute and relative QPCR quantification of plasmid copy number in *Escherichia coli*, *Journal of Biotechnology* **123**: 273-280.

Qi, X., Wang, S., Jiang, Y., Liu, P., Hao, W., Han, J., et al. (2021) Additional polypyrrole as conductive medium in artificial electrochemically active biofilm (EAB) to increase the sensitivity of EAB based biosensor in water quality early-warning, *Biosensors and Bioelectronics* **190**: 113453.

Wang, Y., Wang, L., Huang, W., Zhang, T., Hu, X., Perman, J.A., and Ma, S. (2017) A metal–organic framework and conducting polymer based electrochemical sensor for high performance cadmium ion detection, *Journal of Materials Chemistry A* **5**: 8385-8393.

Xu, J., Zhou, W., Han, X., Liu, J., Dong, Y., Jiang, Y., et al. (2025) Extracellular electron transfer proteins contribute to reduction of ferric minerals by *Geobacter* biofilms, *Applied and Environmental Microbiology* **91**: e00369-00325.

Yang, Y., Ding, Y., Hu, Y., Cao, B., Rice, S.A., Kjelleberg, S., and Song, H. (2015) Enhancing bidirectional electron transfer of shewanella oneidensis by a synthetic flavin pathway, *ACS Synthetic Biology* **4**: 815-823.

Yang, Y., Fang, D., Liu, Y., Liu, R., Wang, X., Yu, Y., and Zhi, J. (2018) Problems analysis and new fabrication strategies of mediated electrochemical biosensors for wastewater toxicity assessment, *Biosensors and Bioelectronics* **108**: 82-88.

Yi, Y., Xie, B., Zhao, T., Qian, Z., and Liu, H. (2019) Effect of control mode on the sensitivity of a microbial fuel cell biosensor with Shewanella loihica PV-4 and the underlying bioelectrochemical mechanism, *Bioelectrochemistry* **128**: 109-117.

Yu, D., Bai, L., Zhai, J., Wang, Y., and Dong, S. (2017) Toxicity detection in water containing heavy metal ions with a self-powered microbial fuel cell-based biosensor, *Talanta* **168**: 210-216.

Yu, Y.-Y., Wang, Y.-Z., Fang, Z., Shi, Y.-T., Cheng, Q.-W., Chen, Y.-X., et al. (2020) Single cell electron collectors for highly efficient wiring-up electronic abiotic/biotic interfaces, *Nature Communications* **11**: 4087.

Zang, Y., Zhao, T., Xie, B., Feng, Y., Yi, Y., and Liu, H. (2021) A bio-electrochemical sensor based on suspended *Shewanella oneidensis* MR-1 for the sensitive assessment of water biotoxicity, *Sensors and Actuators B: Chemical* **341**: 130004.

Zhuang, Z., Yang, G., Mai, Q., Guo, J., Liu, X., and Zhuang, L. (2020) Physiological potential of extracellular polysaccharide in promoting *Geobacter* biofilm formation and extracellular electron transfer, *Science of The Total Environment* **741**: 140365.
